# Effects of basic fibroblast growth factor and cyclin D1 on cigarette smoke-induced pulmonary vascular remodeling in rats

**DOI:** 10.3892/etm.2014.2044

**Published:** 2014-10-31

**Authors:** SIJING ZHOU, MIN LI, DAXIONG ZENG, GENGYUN SUN, JUNSHENG ZHOU, RAN WANG

**Affiliations:** 1 Hefei Prevention and Treatment Center for Occupational Diseases, The First Affiliated Hospital of Anhui Medical University, Hefei, Anhui 230022, P.R. China; 2 Department of Respiratory Medicine, The First Affiliated Hospital of Anhui Medical University, Hefei, Anhui 230022, P.R. China; 3 Department of Oncology, The First Affiliated Hospital of Anhui Medical University, Hefei, Anhui 230022, P.R. China; 4 Department of Respiratory Medicine, The First Affiliated Hospital of Soochow University, Suzhou, Jiangsu 215006, P.R. China

**Keywords:** cigarette smoke, basic fibroblast growth factor, cyclin D1, smooth muscle

## Abstract

Cigarette smoking may contribute to pulmonary hypertension in chronic obstructive pulmonary disease by resulting in pulmonary vascular remodeling that involves pulmonary artery smooth muscle cell proliferation. This study investigated the effects of basic fibroblast growth factor (bFGF) and cyclin D1 on the pulmonary vascular remodeling in smoking-exposed rats. Twenty-four male Wistar rats were randomly divided into four groups. Three tobacco-exposed groups were exposed to the smoke produced by 20 cigarettes for 60 min, twice a day for two, four or eight weeks, and the control group were exposed to fresh air. The expression of bFGF and cyclin D1 in the pulmonary arterial smooth muscle cells were determined using immunohistochemistry. Quantitative polymerase chain reaction was conducted to determine the expression levels of bFGF and cyclin D1 mRNA. In addition, the expression of bFGF and cyclin D1 proteins was evaluated by western blotting. The expression of bFGF and cyclin D1 at the mRNA and protein levels was shown to increase with the duration of smoke exposure (P<0.05). The correlation analysis indicated the expression of bFGF and cyclin D1 was positively associated with the pulmonary vessel wall thickness. The expression of bFGF was positively associated with that of cyclin D1. Collectively, the data demonstrated that the upregulation of bFGF and cyclin D1 occurred in rats subjected to smoke exposure, which may be associated with the abnormal proliferation of the smooth muscle cells in the pulmonary arteries.

## Introduction

Pulmonary vascular remodeling is a significant pathological factor for pulmonary arterial hypertension (1), resulting in increased pulmonary vascular resistance and reduced elasticity. The overproliferation of pulmonary arterial smooth muscle cells is the predominant feature of pulmonary vascular remodeling, which induces thickening of the pulmonary arterial wall, stenosis of the lumina, and muscularization of the pulmonary arteries (2). Previous studies (3,4) have indicated that cigarette smoke induces pulmonary vascular remodeling through direct affects on the lung vessels. However, the potential mechanism remains unclear. Basic fibroblast growth factor (bFGF) has been reported to play an important role in the regulation of fibroblasts, airway smooth muscle cells, and endothelial cells through the autocrine and paracrine systems (5). Nevertheless, to the best of our knowledge, no study has been performed to investigate whether it is involved in the remodeling of lung vessels in rats exposed to cigarette smoke (6).

Cellular proliferation and cell numbers are regulated by the cell cycle, which involves a series of cyclins (7). Cyclin D1 has been shown to play a crucial role in the G1/S transition; for example, Liu and Templeton (8) reported that crocetin inhibited the G1/S transition through the suppression of cyclin D1 in vascular smooth muscle cells. Our previous study (9) indicated that cyclins D1 and E are the rate-limiting activators of the G1/S transition, and that cyclin D1 may play a specialized role in facilitating emergence from quiescence. In the present study, the aim was to investigate the effect of the duration of cigarette smoke exposure on the expression of bFGF and cyclin D1 in the pulmonary vessels in rats, based on which their roles in pulmonary vascular remodeling were investigated.

## Materials and methods

### Animals

A total of 24 male Wistar rats (body weight, 150–200 g; age, 6 weeks) were randomly divided into four groups: Control (n=6), tobacco smoke-exposed I (n=6), tobacco smoke-exposed II (n=6) and tobacco smoke-exposed III (n=6). For the tobacco smoke-exposed groups, the animals were placed in a ventilated smoking chamber and exposed to the smoke produced by 20 cigarettes (nicotine, 1.0 mg per cigarette; tar, 13 mg per cigarette) for 60 min, twice a day for 2 weeks (group I), 4 weeks (group II) and 8 weeks (group III). The control group was exposed to fresh air with no contact with smoke. This study was approved by the Ethics Committee of the First Affiliated Hospital of Anhui Medical University (Hefei, China).

### Sample preparation

The animals were anesthetized with chloral hydrate (10%) and blood (1 ml) was extracted from the abdominal aorta for blood gas analysis prior to sacrifice. The animals were then sacrificed by exsanguination. The chest cavity was opened and the right lung was removed, following which the aortic smooth muscles of the right lobe were separated. Subsequently, the tissues were frozen at −80° for further study. The left lung was perfused with 4% paraform through the trachea until the pleura was flat. The left main bronchus was resected following ligation. Subsequently, the tissues were fixed using 4% paraform and embedded with paraffin.

### Determination of pulmonary vessel wall thickness

Sections were embedded with paraffin and cut to a thickness of 6 μm. Then the sections were stained with hematoxylin and eosin (H&E) as previously described (10). Five pulmonary arteries with a diameter of 50–150 μm were selected using an Olympus BX51 light microscope (Olympus Corporation, Tokyo, Japan). The slides were scanned using an scanner (Canon LIDE110; Canon Inc., Tokyo, Japan). The scanned images were processed using HMIAS-2000 high definition color medical image analysis software (Qianping Co. Ltd., Wuhan, China) to determine the thickness of the pulmonary blood vessels and the pulmonary vessel wall thickness (10). As described in a previous study (9), the pulmonary vessel wall thickness was expressed as the percentage of the external diameter: 2 × measured wall thickness/external diameter × 100.

### Immunohistochemistry

Immunohistochemistry was conducted using the Histostain-SP immunohistochemical staining kit purchased from Beijing Zhongshan Golden Bridge Biological Technology Co., Ltd. (Beijing, China) following the manufacturer’s instructions. In brief, the paraffin sections were incubated overnight at 4°C with mouse anti-rat α-smooth muscle (SM) actin monoclonal antibody (1:200 dilution). The immunohistochemical staining of bFGF and cyclin D1 was conducted in a similar manner as that for α-SM actin, with the exception that a corresponding primary antibody was used. Subsequently, the sections were stained with diaminobenzidine (DAB) and counterstained with hematoxylin (11). The muscularization degree of the arteries was determined according to the staining results (those stained in a buffy color in the intracytoplasm were considered as positive results). The ratio of muscularized arteries was presented as the number of muscularized arteries to the total amount of arterioles.

### Quantitative polymerase chain reaction (qPCR) analysis of the expression of bFGF and cyclin D1 mRNA

Total mRNA was extracted using TRIzol^®^ reagent purchased from Invitrogen Co., Ltd (Shanghai, China) according to the manufacturer’s instructions. cDNA was synthesized using a reverse transcriptase kit (ReverTra Ace-α-, FSK-101; Toyobo, Osaka, Japan), following the manufacturer’s instructions. qPCR was performed using the SYBR^®^ Green Realtime PCR Master Mix (QPK-201, Toyobo) and a Lightcycler^®^ 480 instrument (Roche, Basel, Switzerland). The cDNA fragments were denatured at 95.8°C for 15 sec, annealed at 58.8°C for 15 sec and extended at 72.8°C for 45 sec for 40 cycles. Each sample was examined in triplicate and the mRNA level was normalized by β-actin. The primers used were: for bFGF, 5′-CGTTTGTGCCTATTGTTCTT GTT-3′ and 5′-TGATCCATTGCTTTACCGTCTAC-3′; for cyclin D1, 5′-TGTTCGTGGCCTCTAAGATGAAG-3′ and 5′-GGAAGTGTTCGATGAAATCGTG-3′; and for β-actin, 5′-GACTACCTCATGAAGATCCTG-3′ and 5′-CATAGAGGTCTTTACGGATGT-3′: The amplification results for qPCR was calculated as 2(^−ΔΔCt^), where ΔΔCt = (Ct gene of interest − Ct control) − (Ct control − Ct control).

### Western blot analysis

For the determination of bFGF and cyclin D1, western blot analysis was performed as previously described (9). Briefly, the tissues were homogenized in radioimmunoprecipitation assay lysis buffer containing protease and phosphatase inhibitors. Proteins were separated by electrophoresis on a 10% SDS-PAGE gel and transferred to a Hybond-P polyvinylidene difluoride membrane. Subsequently, the membrane was blocked in 5% non-fat milk and incubated with a primary antibody (rabbit anti-bFGF or rabbit anti-cyclin D1 antibody; 1:1,000 dilution; Santa Cruz Biotechnology, Inc., Santa Cruz, CA, USA) overnight at 4°C, followed by incubation with the peroxidase-conjugated secondary antibody [rabbit anti-goat immunoglobulin G (IgG) or goat anti-rabbit IgG; 1:3,000 dilution; Santa Cruz Biotechnology, Inc.] for 1 h at room temperature. Subsequent to washing with phosphate-buffered saline, the bound primary antibody was visualized and exposed to X-ray film. The same membrane was probed for β-actin as the loading control. The analyses of cyclin D1 and bFGF were conducted in a similar manner, with the exception of the primary and secondary antibodies. The relative density of bFGF and cyclin D1 to that of β-actin was analyzed with Quantity One software (Bio-Rad, Hercules, CA, USA).

### Statistical analysis

All data are presented as the mean ± standard error of the mean. SPSS software, version 12.0 (SPSS, Inc., Chicago, IL, USA) was used for the statistical analyses. χ^2^ test was performed for inter-group comparisons, and analyzed with one-way analysis of variance. Pearson correlation analysis was used to analyze the correlations between the percentage wall thickness (%WT), and bFGF and cyclin D1 mRNA and protein in the pulmonary artery smooth muscle cells. P<0.05 was considered to indicate a statistically significant result in all statistical analyses.

## Results

### Partial pressure of oxygen in arterial blood (PaO_2_)

Table I summarizes the PaO_2_, %WT and the muscularization of the pulmonary arteries. No statistical difference was noted in the PaO_2_ among the four groups (P>0.05).

### H&E staining and %WT

**Figure 1:**
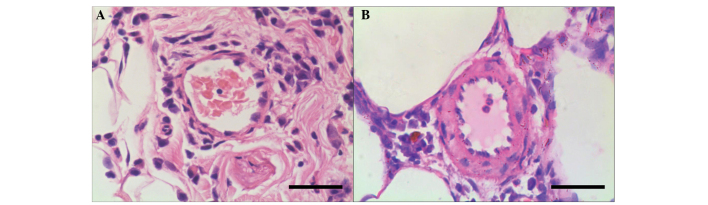
Pulmonary arteries of rats in (A) the control group and (B) the eight weeks smoke exposure group (magnification, ×400; scale, 20 μm; hematoxylin and eosin staining).

No abnormality was noted in the anatomical structure of the lung in the control group (Fig. 1A). Gradual thickening of the pulmonary vascular wall was noted. No significant signs of emphysema, such as anatomical disorder of the alveolus and dilatation of alveolar space, were observed. Vessel wall thickness exhibited a significant increase in the smoke exposure group as compared with the control group (Fig. 1B). Compared with the control group, the %WT showed significant increases in the three smoking exposure groups. Statistically significant differences were noted in the %WT subsequent to the animals being exposed to smoke for two, four and eight weeks, respectively (Table I).

### Pulmonary arteriole muscularization

With regard to the muscularization of the pulmonary arteriole, notable increases were identified in the smoke exposure groups compared with that of the control group (P<0.05). Enhanced pulmonary arteriole muscularization was noted with the increase of the exposure duration (Table I).

### Immunohistochemistry, qPCR and western blotting

**Figure 2:**
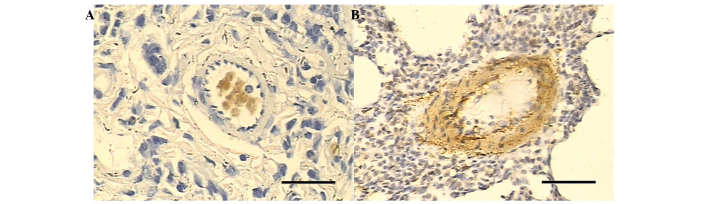
Immunohistochemical staining of basic fibroblast growth factor in the rats in (A) the control group and (B) the eight weeks smoke exposure group (magnification, ×400; scale, 20 μm).

**Figure 3:**
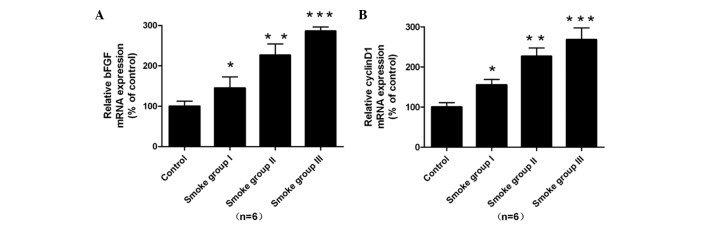
bFGF and cyclin D1 mRNA levels were determined by quantitative polymerase chain reaction, normalized against β-actin. Results are presented as percentages of the expression in the control group. Data are presented as the means ± standard error of the mean from six independent experiments. (A) The relative expression of bFGF mRNA in each group. (B) The relative expression of cyclin D1 mRNA in each group. ^*^P<0.05 vs. the control group, ^**^P<0.05 vs. smoke-exposed group I and ^***^P<0.05 vs. smoke-exposed group II. bFGF, basic fibroblast growth factor.

No positive staining of bFGF was identified in the pulmonary arterial smooth muscle cells in the control group (Fig. 2A). Low staining of bFGF was noted in the pulmonary arterial smooth muscle cells of the smoke-exposed group I. Enhanced staining of bFGF was noted in the smoke-exposed groups II and III compared with that of the smoke-exposed group I (Fig. 2B). With regard to the expression of mRNA, upregulation of bFGF mRNA was noted in the smooth muscle cells in the smoke-exposed groups compared with that of the normal control (P<0.05, Fig. 3A).

**Figure 4:**
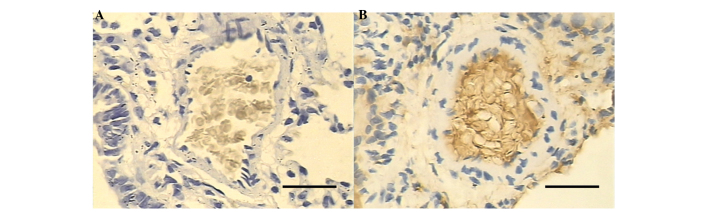
Cyclin D1 immunohistochemical staining. of rats in (A) the control group, (B) the eight weeks smoke exposure group (magnification, ×400; scale, 20 μm).

**Figure 5:**
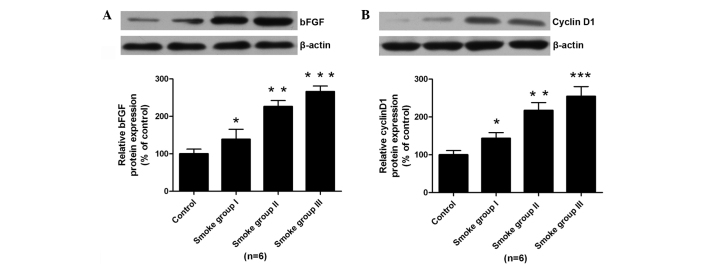
bFGF and cyclin D1 protein levels were determined by western blotting, normalized against β-actin. The graph shows the results of the densitometric quantification of the western blot analysis, which are presented as percentages of the control group. Data are presented as the mean ± standard error of the mean from six independent experiments. (A) The relative expression of bFGF protein in each group. (B) The relative expression of cyclin D1 protein in each group. ^*^P<0.05 vs. the control group. ^**^P<0.05 vs. smoke group I. and ^***^P<0.05 versus smoke group II. bFGF, basic fibroblast growth factor.

With regard to the positive immunohistochemical staining of cyclin D1, no staining was noted in the control group (Fig. 4A), while the smoke-exposed groups I and II showed moderate increases compared with that of the control group. Enhanced staining of cyclin D1 was observed in the smoke-exposed group III (Fig. 4B). Furthermore, enhanced expression of cyclin D1 mRNA was observed in the smoke-exposed groups compared with that in the normal control (P<0.05, Fig. 3B). With regard to the protein expression levels of bFGF and cyclin D1 determined by western blotting, upregulation of bFGF and cyclin D1 was noted in the smoke-exposed groups compared with that of control group (P<0.05, Fig. 5).

### Correlation analysis

Pearson’s correlation analysis indicated that the %WT was positively associated with the expression of bFGF mRNA (r=0.907, P<0.01) and bFGF protein (r=0.912, P<0.01) in the pulmonary arterial smooth muscle cells. In addition, the %WT was positively with the expression of cyclin D1 mRNA (r=0.918, P<0.01) and cyclin D1 protein (r=0.906, P<0.01). Furthermore, the mRNA expression of bFGF and cyclin D1 was positively associated with that of bFGF protein (r=0.914, P<0.01) and cyclin D1 protein (r=0.922, P<0.01), respectively.

## Discussion

Pulmonary hypertension denotes a disorder of the pulmonary arterioles that is characterized by elevated pulmonary artery pressure, right-heart failure, persistent vasoconstriction, thickening of the pulmonary vascular wall, vascular remodeling and a progressive increase of pulmonary vascular resistance (12,13). Generally, the pathological foundation of pulmonary hypertension includes muscularization of the pulmonary arteries induced by the overproliferation and migration of pulmonary smooth muscle cells, intima-media thickening of the arteriole and neointimal formation. Smoking has been considered as a great threat to public health as it is a risk factor for a variety of diseases. Cigarettes contain >4,000 chemical compounds, and ≥400 toxic substances including nicotine, tar, acrolein, carbon monoxide, hydrogen cyanide, ammonia, aromatic compounds, arsenic, mercury and chromium. The pulmonary vascular structure in smokers has been extensively investigated, and has shown that cigarette smoke is able to induce the remodeling of pulmonary vessels (14). In the present study, the percentage of pulmonary arteriole muscularization showed a marked increase following exposure to cigarette smoke for four and eight weeks compared with that in the control group, which demonstrated that pulmonary vascular remodeling was induced following exposure to cigarette smoke. As no reduction in PaO_2_ was observed, it may be speculated that cigarette smoke was involved in the pulmonary vascular remodeling through direct interaction with the pulmonary vasculature. The present study was consistent with a previous study (15).

Previous studies have indicated that the upregulation of the gene expression and protein production of vascular cell proliferative agents, endothelin and vascular endothelial growth factor is associated with the pulmonary vascular remodeling induced by cigarette smoke (16,17). bFGF is a potent regulator of various cellular functions, including mitosis, cell proliferation, differentiation, survival, cellular adhesion, migration, motility and apoptosis, vasculogenesis and blood vessel remodeling (18–20). In the present study, the expression of bFGF was identified in the pulmonary arteries following exposure to cigarette smoke. Also, the upregulation of bFGF mRNA and protein was noted. Muscularization of the pulmonary artery and %WT were found to be positively correlated with the expression of bFGF mRNA and protein. These results indicate that bFGF plays a crucial role in the pulmonary arterial remodeling induced by cigarette smoke, which is consistent with the findings of previous studies (21,22).

The present results showed that upregulation of cyclin D1 mRNA and protein occurred in the smooth muscle cells of the pulmonary artery in rats exposed to cigarette smoke. Additionally, the expression of cyclin D1 mRNA and protein was found to be positively correlated with the muscularization of the pulmonary artery and %WT. In addition, the expression levels of cyclin D1 mRNA and protein were positively correlated with those of bFGF. Based on these results, it may be speculated that the smoke exposure induced the upregulation of bFGF, which resulted in the upregulation of cyclin D1 subsequently. Following this, proliferation of the smooth muscle cells in the pulmonary artery was triggered due to the upregulation of cyclin D1, resulting in pulmonary vascular remodeling.

In conclusion, the present study demonstrates that the upregulation of bFGF and cyclin D1 occurs in the smooth muscle cells of the pulmonary arteries following exposure to cigarette smoke and is associated with the duration of cigarette smoke exposure. The expression levels of bFGF mRNA and protein were found to be positively correlated with those of cyclin D1, which indicates that these two proteins play a crucial role in the remodeling of the pulmonary artery. The present study may provide helpful information for the identification of treatment targets for pulmonary hypertension. Further studies are required to investigate the potential roles of bFGF and cyclin D1 in the process of pulmonary arterial remodeling.

## Figures and Tables

**Table I:** PaO_2_, %WT and the muscularization of the pulmonary arteries.

Group	PaO_2_ (mmHg)	%WT	Muscularization (%)
Control	91±4.5	20.5±1.8	4.1±1.2
Smoke-exposed I	92±4.6	31.6±1.4^a^	6.2±1.8
Smoke-exposed II	91±3.7	39.3±2.1^a^,^b^	17.5±2.6^a^,^b^
Smoke-exposed III	93±4.1	50.7±1.5^a^–^c^	28.3±4.5^a^–^c^

Data are presented as the mean ± standard error of the mean.

a P<0.05 vs. control group,

b P<0.05 vs. smoke-exposed group I,

c P<0.05 vs. smoke-exposed group II.

PaO_2_, oxygen pressure in arterial blood; %WT, pulmonary vessel wall thickness expressed as the percentage of the external diameter (2 × measured wall thickness/external diameter ×100).
